# Substance Use Patterns and Their Association with Depression and Social Factors During COVID-19 Among Harlem Residents in New York City

**DOI:** 10.1007/s10900-023-01253-1

**Published:** 2023-07-07

**Authors:** Thinh T. Vu, Joseph P. Dario, Pedro Mateu-Gelabert, Deborah Levine, Malcolm A. Punter, Luisa N. Borrell, Victoria K. Ngo

**Affiliations:** 1 Center for Innovation in Mental Health, Graduate School of Public Health & Health Policy, The City University of New York, New York, USA; 2 Department of Community Health and Social Sciences, Graduate School of Public Health & Health Policy, The City University of New York, New York, USA; 3 Division of Pediatric Critical Care, Mount Sinai Kravis Children’s Hospital, New York, USA; 4 Harlem Health Initiative, Graduate School of Public Health & Health Policy, The City University of New York, New York, USA; 5 Harlem Congregations for Community Improvement, Inc., New York, USA; 6 Department of Epidemiology and Biostatistics, Graduate School of Public Health & Health Policy, The City University of New York, New York, USA

**Keywords:** Substance use, Depression, Housing insecurity, Employment insecurity, Community perception of police, New York City

## Abstract

This study aimed to identify the prevalence of substance use before and during COVID-19; and examined its association with depression and social factors among 437 residents from the neighborhood of Harlem in Northern Manhattan, New York City. Over a third of respondents reported using any substance before COVID-19, and initiating/increasing substance use during COVID-19. The most common substances used before COVID-19 and initiated/increased during COVID-19 were smoking (20.8% vs. 18.3%), marijuana (18.8% vs. 15.3%), and vaping (14.2% and 11.4%). The percentages of any hard drug use were 7.3% and 3.4%, respectively. After adjustment, residents with mild (Prevalence Ratio [PR] = 2.86, 95% CI 1.65, 4.92) and moderate (PR = 3.21, 95% CI 1.86, 5.56) symptoms of depression, and housing insecurity (PR = 1.47, 95% CI 1.12, 1.91) had at least a 47% greater probability of initiating and/or increasing substance use. Conversely, respondents with employment insecurity (PR = 0.71, 95% CI 0.57, 0.88) were 29% less likely to report such patterns. No association was found between substance use initiation and/or increase and food insecurity. High prevalence of substance use during COVID-19 may lead residents to turn to substance use as a coping mechanism for psychosocial stressors. Thus, it is essential to provide accessible and culturally sensitive mental health and substance use services.

## Introduction

Substance use has been a major public health issue leading to drug overdoses, disease transmission, and contributing to the mental health burden in the USA. [1]. This issue has been exacerbated by COVID-19, with 13.3% of Americans reporting initiating or increasing substance use as a coping mechanism for stress and/or emotions associated with COVID-19 after June 2020 [2]. Parallel to this increase in substance use, there was an 18% increase in drug overdoses nationwide within the first several months of the pandemic compared to those same months in 2019 [3]. Notably during COVID-19, Black Americans who engaged in substance use and contracted COVID-19 had higher mortality and rate of hospitalization than white Americans [4]. Within Manhattan in New York City (NYC), Black residents had the highest rate of overdose-related deaths in 2021 (77.6%), followed by Hispanic/Latino residents (48.6%), mostly due to cocaine, fentanyl, and heroin [5]. The initiation and/or increase of substance use within minoritized communities during COVID-19 is not well described, especially in Harlem, a neighborhood in Northern Manhattan, NYC, where the population is predominantly Black, hindering the understanding of substance use needs in vulnerable communities.

Since the Great Depression in 1929, COVID-19 caused a major economic fallout in NYC leading to the highest level of unemployment at 13.2% in October 2020, an estimate close to double the national unemployment rate (6.9%). This led to a reduction in salary income, food insecurity, and the ability to pay housing obligations [6, 7]. Additionally, people who work from home were coping with challenges because of school closures and a lack of childcare [7]. Coping with the double crisis in terms of health conditions and the economy, Americans witnessed extremely high rates of anxiety and depression disorders ranging between 30 and 56% [8–12]. The deleterious toll on mental health and substance use was observed but little is known about their relationship during the COVID-19 pandemic [13]. While the stressors from COVID-19 impacted and continue to impact people throughout the city disproportionally across race and ethnicity, they are predominantly prevalent among low- and middle-income Black and Hispanic/Latino populations in Harlem [14, 15]. Nonetheless, current literature has not fully examined the association between substance use initiation and/or increase and social factors such as employment insecurity, housing insecurity, food insecurity, as well as childcare challenges during COVID-19 [13].

More importantly, previous research focused on specific populations such as college students, and convicted felons [16, 17], therefore, much research is needed for substance use in the general population [18, 19], especially among heavily-impacted communities in urban settings. Also, no studies have examined the impact of public perception of police on substance use during COVID-19. However, low perception of police might be associated with higher substance use initiation and/or increase during the pandemic. This study aimed to identify the prevalence of substance use before, and after (i.e., initiation and/or increase) the first NYC case of COVID-19 was reported in March 2020; and examined its association with depression severity and social factors among Harlem residents in Northern Manhattan.

## Methodology

### Study Setting

This study was conducted in the Harlem neighborhood, located in the northern section of the borough of Manhattan in New York City, USA. Harlem is one of NYC’s poorest neighborhoods with over one-fifth of all households living below the federal poverty level. Moreover, Harlem has historically been under-served, with 13% of all adult residents lacking health insurance and 1 in 9 Harlem adult residents going without needed medical care [20–22].

### Study Design and Sample Size

From April to September 2021, this cross-sectional study employed a Qualtrics platform to recruit self-reported Harlem residents who were at least 18 years old. A meticulous approach was adopted to ensure data quality, as detailed elsewhere [23], encompassing open-ended queries and honeypot questions with JavaScript programming to mitigate the impact of automated bots and spam responses. Furthermore, all participants were verified by cross-referencing their responses through email correspondence, phone conversations, and by consulting the online directory, Whitepages.com, leading to a sample size of 437 participants.

### Measurements

#### Substance Use

The survey evaluated the self-reported history of substance use before March 2020, including nine specific types of drugs: alcohol, marijuana, vaping, smoking, cigarettes, hard drugs (including crack, cocaine, and heroin), and non-medical use of prescription medication (also known as prescription drug misuse). Additionally, participants were asked whether they initiated and/or increased their usage of any drugs after March 2020. Alcohol use was excluded from this study as it was examined extensively in a separate study [23].

#### Depression Severity

We administered the Patient Health Questionnaire with 4 items (PHQ-4) to evaluate depression symptomatology during COVID-19 [24]. Participants responded to the questions using a 3-point Likert scale describing frequency of depression symptoms within the past 2 weeks, ranging from 0 (Not at all) to 3 (Nearly every day). A summary score ranged from 0 to 12 with depression severity categorized as none (0–2), mild (3–5), moderate (6–8), and severe (9–12). The PHQ-4 demonstrated good internal consistency, with a Cronbach’s alpha of 0.8 in this study [23].

#### Social Risk Factors

This study incorporated four binary domains on the social impact of COVID-19 [23], as follows: (1) *Employment insecurity*: Participants who were either unemployed, worked part-time intermittently, or did not receive payment during periods of unemployment; (2) *Housing insecurity*: Participants currently living in a shelter, facing eviction, or experiencing difficulties paying rent or mortgage; (3) *Food insecurity*: Participants who frequently or occasionally did not have enough to eat or struggled to afford additional food; and (4) *Childcare challenges*: Participants facing difficulties in childcare or unable to work due to caring for children not attending school or daycare among respondents who had children (n = 265).

#### Interpersonal Violence

The Humiliation, Afraid, Rape, Kick (HARK) questionnaire [25] with 4 binary questions were used to evaluate exposure to violence within the household since the beginning of the pandemic. For instance, participants were asked if they had experienced humiliation or emotional abuse by anyone in their household or if they had feared harm from someone in their household. A positive response to any of the 4 items indicated the presence of interpersonal violence. The HARK scale demonstrated good internal consistency, with a Cronbach’s alpha of 0.70 in our cohort [23].

#### Community Perceptions of Police

To assess how respondents viewed the police force, a 10-item questionnaire was developed [23]. Each item presented statements such as “*I feel safer when I see police presence in my community*” or “*The police have been respectful and responsive to the needs of demonstrators*”. Participants rated their agreement on a scale of 1 (Strongly disagree) to 5 (Strongly agree), with a total score ranging from 10 to 50, where higher scores indicated a higher level of police satisfaction.

#### Demographics

Characteristics included were age in years, gender (male, female, and other), race/ethnicity (white vs. non-white), place of birth (born in the U.S. vs. no), current marital status (yes vs. no), household size including the participant (1, 2, or 3–8 people), educational attainment (high school or less, associate’s or some college degree, bachelor’s or graduate degree), employment status (unemployed vs. employed), work changed during COVID-19 (yes vs. no), and respondents’ annual income (< $25 K, $25–$49 K, and ≥ $50 K).

### Statistical Analysis

Continuous variables were summarized using mean and standard deviation (SD), while categorical variables were reported as frequencies and percentages. To test the associations between demographics, depression and social risks, and initiation and/or increase of substance use, *t*-tests, Fisher’s exact tests, and Chi-squared analyses were used. Given the high prevalence of substance use initiation and/or increase, multivariable log-binomial regression analyses were performed, to examine the association between demographic factors, depression severity and social factors, and initiation and/or increase of substance use, after adjusting for age, gender, and race/ethnicity. Prevalence ratios (PRs) and confidence intervals (CIs) were reported. Data management and analyses were conducted using STATA version 17.

## Results

### Demographic Characteristics and Substance Use Initiation and/or Increase

Among 437 Harlem residents, the average age was 34.2 (SD = 8.7) with 47.4% falling between 30 and 39 years old; more than half were female (52.2%) and two-thirds were non-white (65.4%). The majority of participants (90.8%) were born in the U.S., and about two-thirds (67.7%) were married. Most respondents lived with 3–8 people in the household (77.7%) and achieved at least an associate’s or some college degree (70.7%). Up to 18.1% experienced unemployment and 68.9% had work changes during COVID-19. More than half (55%) reported an annual income ranging from $25 K to $49 K (Table 1).

**Table 1:** Demographics and its association with substance use initiation and/or increase in Harlem residents: 2021

	Total	Substance use initiation and/or increase		p-value^a^
Characteristics		No	Yes	
	N = 437	n = 268	n = 169	
Age, years (mean ± SD)	34.2 ± 8.7	35.9 ± 8.7	31.7 ± 8.3	< 0.001
Age groups				< 0.001
18–29	133 (30.4)	59 (22.0)	74 (43.8)	
30–39	207 (47.4)	137 (51.1)	70 (41.4)	
40–81	97 (22.2)	72 (26.9)	25 (14.8)	
Gender				< 0.001
Female	228 (52.2)	167 (62.3)	61 (36.1)	
Male	170 (38.9)	76 (28.4)	94 (55.6)	
Others	39 (8.9)	25 (9.3)	14 (8.3)	
Ethnicity				0.26
White	151 (34.6)	87 (32.5)	64 (37.9)	
Not white	286 (65.4)	181 (67.5)	105 (62.1)	
Born outside of the United States				0.24
No	397 (90.8)	247 (92.2)	150 (88.8)	
Yes	40 (9.2)	21 (7.8)	19 (11.2)	
Being currently married				0.006
No	141 (32.3)	73 (27.2)	68 (40.2)	
Yes	296 (67.7)	195 (72.8)	101 (59.8)	
Current household size				0.51
Only participant	43 (9.8)	30 (11.2)	13 (7.7)	
Participant and one other	72 (16.5)	44 (16.4)	28 (16.6)	
3–8 people	322 (73.7)	194 (72.4)	128 (75.7)	
Educational attainment				0.85
High school and less	128 (29.3)	81 (30.2)	47 (27.8)	
Associate’s or some college’s degree	229 (52.4)	138 (51.5)	91 (53.9)	
Bachelor or graduate degree	80 (18.3)	49 (18.3)	31 (18.3)	
Respondents’ unemployment status				< 0.001
Employed	358 (81.9)	195 (72.8)	163 (96.4)	
Unemployed	79 (18.1)	73 (27.2)	6 (3.6)	
Having work changed during COVID-19				0.34
No	136 (31.1)	88 (32.8)	48 (28.4)	
Yes	301 (68.9)	180 (67.2)	121 (71.6)	
Respondents’ yearly income^b^				< 0.001
< $25 K	115 (26.7)	57 (21.8)	58 (34.3)	
$25–$49 K	237 (55.0)	166 (63.4)	71 (42.0)	
≥ $50 K	79 (18.3)	39 (14.9)	40 (23.7)	

^a^*T*-test and Chi-squared tests

^b^Refused to answer (n = 6)

In bivariate analyses, several demographic factors were found to be associated with the initiation and/or increase of substance use during COVID-19, including younger age, male, currently married, unemployed, and having a higher annual income (all p-values < 0.01).

### History of Substance Use Before COVID-19 and Substance Use Initiation and/or Increase During COVID-19

**Fig. 1:**
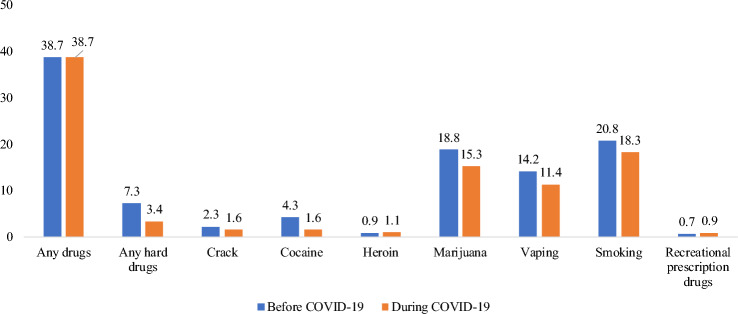
History of substance use before COVID-19 (before March 2020) and substance use initiation and/or increase during COVID-19 (after March 2020)

Over a third of respondents reported using any substance before COVID-19 and initiating/increasing substance use during COVID-19 (38.7% for both time periods; Fig. 1). The top three substances used before COVID-19 and initiated/increased during COVID-19, respectively, were smoking (20.8% vs. 18.3%), marijuana (18.8% vs. 15.3%), and vaping (14.2% and 11.4%). The corresponding estimates for hard drug use before and during COVID-19 were 7.3% and 3.4%. Less than 1% reported prescription drug misuse.

### Depression, Social Factors, and Substance Use Initiation and/or Increase

Factors associated with substance use initiation and/or increase during COVID-19 included higher severity of depression, experiencing interpersonal violence, employment insecurity, housing insecurity, and food insecurity. Conversely, those who reported higher satisfaction with police were less likely to report substance use initiation and/or increase (Table 2). Those who experienced childcare challenges were not associated with substance use initiation and/or increase.

**Table 2:** Depression and social factors and their associations with substance use initiation and/or increase in Harlem residents: 2021

	Total^a^	Substance use initiation and/or increase		p-value^b^
		No	Yes	
	N = 437	n = 268	n = 169	
PHQ-4 summary score (mean ± SD)	4.5 ± 2.8	3.9 ± 3.0	5.5 ± 2.1	< 0.001
Depression severity				< 0.001
None	118 (27.0)	105 (89.0)	13 (11.0)	
Mild	158 (36.2)	82 (51.9)	76 (48.1)	
Moderate	119 (27.2)	53 (44.5)	66 (55.5)	
Severe	42 (9.6)	28 (66.7)	14 (33.3)	
Interpersonal violence				< 0.001
No	152 (34.8)	112 (73.7)	40 (26.3)	
Yes	285 (65.2)	156 (54.7)	129 (45.3)	
Employment insecurity				0.001
No	192 (43.9)	101 (52.6)	91 (47.4)	
Yes	245 (56.1)	167 (68.2)	78 (31.8)	
Housing insecurity				< 0.001
No	204 (46.7)	153 (75.0)	51 (25.0)	
Yes	233 (53.3)	115 (49.4)	118 (50.6)	
Food insecurity				< 0.001
No	237 (54.2)	170 (71.7)	67 (28.3)	
Yes	200 (45.8)	98 (49.0)	102 (51.0)	
Childcare challenge^c^				0.16
No	124 (46.8)	86 (69.4)	38 (30.6)	
Yes	141 (53.2)	86 (61.0)	55 (39.0)	
Satisfaction with police (10–50)	25.9** ± **4.3	26.2** ± **4.5	25.3** ± **4.1	0.049

*PHQ-4* Patient Health Questionnaire with 4 items

^a^Percentages by column

^b^*T*-test and Chi-squared tests

^c^Among individuals with children (n = 265)

### Associations Between Demographic, Depression, Social Factors, and Substance Use Initiation and/or Increase

After controlling for age, gender, and race/ethnicity (Table 3), residents with mild (Adjusted prevalence ratio [aPR] = 2.86, 95% CI 1.65, 4.92) and moderate (aPR = 3.21, 95% CI 1.86, 5.56) symptoms of depression had at least a 186% higher probability of initiating and/or increasing substance use during the pandemic than residents without depression. No significant association was found between severe depression and substance use initiation and/or increase. Participants who reported employment insecurity (aPR = 0.71, 95% CI 0.57, 0.88) were 29% less likely to initiate and/or increase substance use, while those who reported housing insecurity were 47% more likely to do so (aPR = 1.47, 95% CI 1.12, 1.91). There was no significant association between food insecurity and substance use initiation and/or increase.

**Table 3:** Adjusted prevalence ratios (aPR) and their 95% confidence intervals (CI) for the associations of depression and social stressors with substance use initiation and/or increase in Harlem residents: 2021

	aPR	95% CI
Depression symptomatology (vs. none)		
Mild	2.86	1.65, 4.92
Moderate	3.21	1.86, 5.56
Severe	1.97	0.99, 3.91
Employment insecurity (vs. no)	0.71	0.57, 0.88
Food insecurity (vs. no)	1.25	0.97, 1.61
Housing insecurity (vs. no)	1.47	1.12, 1.91

The model was controlled for gender (male, female, vs. other), race/ethnicity (white vs. non-white), and age (in years)

The estimates from the fully-adjusted model showed minimal change when social-economic status (e.g., if born outside the U.S., education levels, and participant’s annual income) and two social factors (e.g., interpersonal violence and community perception of police) were included. Additionally, childcare challenge was not included because only 265 residents reported having children. Therefore, these variables were excluded from the final model for parsimony purposes.

## Discussion

In this study, we observed a high percentage of Harlem residents reporting initiating and/or increasing substance use during COVID-19, at 38.7%. Our finding aligns with a recent study in the general population across 16 countries [18], but more than double the corresponding estimate reported in the 2020 survey, where only 13% of adult Americans started or increased substance use as a means of coping with stress or emotions related to COVID-19 [26]. These differences could be influenced by variations in the populations considered, as our study focused on a community primarily comprised of Black individuals, who have been disproportionately affected by the pandemic in NYC [23]. Moreover, as a major epicenter of the pandemic in 2020, NYC may have experienced a more profound impact from the pandemic on substance use and initiation than the rest of the country. During COVID-19, the initiation and/or increase of substance use can be attributed to multiple factors. A possible reason could be the need to handle social stress and uncertainty that accompanied the global health crisis. With the disruption of daily routines, social isolation, and concerns about health and financial stability, individuals may have turned to substance use as a way to manage their anxiety and emotional distress [18]. Notably, smoking, marijuana, and vaping were among the substances most commonly initiated or increased during this period, which is consistent with previous studies [27–29]. This pattern could be partly attributed to the ease of accessibility of these substances, especially considering the legal status of marijuana for adult use in New York State since March 2021 [29]. Therefore, it is crucial to raise awareness about the detrimental effects of smoking, vaping, and marijuana, as well as promote smoking cessation as a means to break this unhealthy habit.

A systematic review showed that mental health factors were identified as the predominant correlates or triggers for increased use of both alcohol and other substances [18], with COVID-19-associated worry and fear specifically impacting substance use increase and initiation [2, 13]. There is a frequent co-occurrence of mental health conditions and substance use disorders. Particularly, among individuals with alcohol and drug dependence, the prevalence of mental health disorders were 30% and 45%, respectively, compared to non‐dependent individuals (12%) [30]. However, the treatment gap was large, with only 1 in 5 people experiencing major depressive disorders receiving minimally adequate treatment in high-income countries, including the USA. [31]. Addressing both mental health and substance misuse simultaneously is crucial to achieving optimal health outcomes, as these individuals have poorer outcomes and more treatment complications than those with substance use issues alone [32, 33]. Integrated approaches that acknowledge the interconnected nature of these problems and treat each problem appropriately are necessary [33]. By offering comprehensive care that targets mental health and substance use concurrently, individuals have a better chance of recovery and improved overall health and well-being.

Our study findings revealed a higher likelihood of individuals experiencing housing insecurity to initiate and/or escalate substance use during COVID-19, possibly due to the significant stress caused by being unstably housed [34]. Hence, it is important to address the housing needs of people who use substances, particularly in metropolitan areas like NYC. Interestingly, individuals who experienced employment insecurity were less likely to initiate and/or increase substance use during COVID-19. Although both housing insecurity and employment insecurity should similarly impact stress experienced by the individuals [35], employment insecurity may limit these individuals from access to substances or may push individuals to avoid drugs to seek stable jobs. Another possibility is that a federal law implemented in 1996 removed alcoholism and addiction as qualifying grounds for benefit claims such as Social Security Disability Insurance or Supplemental Security Income [36]. However, if the Social Security Administration determines that an individual’s drug or alcohol use directly causes or contributes to the physical or functional limitations that render them from working, their benefit claim may be rejected. Consequently, participants may exhibit a reduced willingness to disclose their substance use due to concerns about the potential impacts on their benefits. Investigating factors behind reluctance to disclose substance use can inform more effective interventions, tailored to the unique needs of individuals facing housing and employment insecurity, fostering inclusive and supportive environments.

We did not observe an association between food insecurity and initiation and/or increase of substance use. However, previous studies showed that there might exist a bidirectional relationship between substance use and food insecurity. For example, allocating limited resources to substance use could compromise the ability to pay for food [37, 38]. Therefore, providing a referral to food resources among those who suffer from food insecurity (e.g., Supplemental Nutrition Assistance Program) is needed to break the pathway from substance use leading to mental health. Similarly, no association was found between police perception and substance use initiation and/or increase. This may be due to difficulty evaluating specific drug classes in this population, as our analysis focused on any substance use initiation and/or increase. Further exploration would be beneficial to understand the reasons behind these findings and specifically understand how specific drug class use may be associated with varying degrees of police satisfaction.

This study has several limitations that warrant discussion. In addition to the limitations mentioned elsewhere [23], such as the issue of external validity due to the study being conducted in a community with a predominantly Black population, the presence of social desirability bias, and low internal consistency of the community perception of police scale, there are additional limitations specific to this paper. It is also difficult to compare the prevalence of substance use before and during COVID-19, as our questionnaire only asked about initiation and/or escalation of substance use at the second timepoint. Another limitation is the lack of data on the frequency and dosage of substance use since we did not employ standardized scales such as The Alcohol, Smoking, and Substance Involvement Screening Test (ASSIST) [39]. Despite this limitation, our findings emphasized the concerning nature of substance use among residents of Harlem. Finally, the sample size in our study was small. As a result, we grouped smoking, vaping, recreational prescription drugs, and hard drugs as a composite variable representing substance use in the final model. However, future research should undertake comparative analyses specifically examining different drug classes to enhance our understanding of the nuanced distinctions between various types of drug use.

## Conclusions

The high prevalence of substance use initiation and/or increase during COVID-19 suggests that Harlem residents may have resorted to substance use as a coping mechanism to navigate the challenges of depression and social stressors. This finding underscores the intricate interplay between depression, social factors, substance use, and the pandemic itself. It emphasizes the critical need to prioritize mental health support and address the underlying social circumstances to gain a comprehensive understanding of substance use trends and effectively mitigate their impacts. By taking into account these multifaceted dynamics, we can develop tailored interventions and culturally sensitive support systems that address the root causes and promote healthier coping strategies during global health crises.
