# Correction: Substance Use Patterns and Their Association with Depression and Social Factors During COVID-19 Among Harlem Residents in New York City

**DOI:** 10.1007/s10900-023-01313-6

**Published:** 2023-12-02

**Authors:** Thinh T. Vu, Joseph P. Dario, Pedro Mateu-Gelabert, Deborah Levine, Malcolm A. Punter, Luisa N. Borrell, Victoria K. Ngo

**Affiliations:** 1 Center for Innovation in Mental Health, Graduate School of Public Health & Health Policy, The City University of New York, New York, USA; 2 Department of Community Health and Social Sciences, Graduate School of Public Health & Health Policy, The City University of New York, New York, USA; 3 Division of Pediatric Critical Care, Mount Sinai Kravis Children’s Hospital, New York, USA; 4 Harlem Health Initiative, Graduate School of Public Health & Health Policy, The City University of New York, New York, USA; 5 Harlem Congregations for Community Improvement, Inc, New York, USA; 6 Department of Epidemiology and Biostatistics, Graduate School of Public Health & Health Policy, The City University of New York, New York, USA

**Journal of Community Health (2023) 48:937?944**

10.1007/s10900-023-01253-1

The article ‘‘Substance Use Patterns and Their Association with Depression and Social Factors During COVID-19 Among Harlem Residents in New York City’’, written by Thinh T. Vu, Joseph P. Dario, Pedro MateuGelabert, Deborah Levine, Malcolm A. Punter, Luisa N. Borrell and Victoria K. Ngo was originally published electronically on the publisher’s internet portal on 07 July 2023 without open access. With the author(s) decision to opt for Open Choice, the copyright of the article changed on 5 October 2023 to The Author(s) 2023 and the article is forthwith distributed under a Creative Commons Attribution 4.0 International License, which permits use, sharing, adaptation, distribution and reproduction in any medium or format, as long as you give appropriate credit to the original author(s) and the source, provide a link to the Creative Commons licence and indicate if changes were made. The images or other third-party materials in this article are included in the articles Creative Commons licence, unless indicated otherwise in a credit line to the material. If material is not included in the articles Creative Commons licence and your intended use is not permitted by statutory regulation or exceeds the permitted use, you will need to obtain permission directly from the copyright holder. To view a copy of this licence, visit http://creativecommons.org/licenses/by/4.0.

